# Seeing the unseen of Chinese herbal medicine processing (*Paozhi*): advances in new perspectives

**DOI:** 10.1186/s13020-018-0163-3

**Published:** 2018-01-17

**Authors:** Xu Wu, Shengpeng Wang, Junrong Lu, Yong Jing, Mingxing Li, Jiliang Cao, Baolin Bian, Changjiang Hu

**Affiliations:** 1 Laboratory of Molecular Pharmacology, Department of Pharmacology, School of Pharmacy, Southwest Medical University, Luzhou, Sichuan China; 2 State Key Laboratory of Quality Research in Chinese Medicine, Institute of Chinese Medical Sciences, University of Macau, Macao, China; 3 College of Pharmacy, Chengdu University of Traditional Chinese Medicine, Liutai Avenue, Wenjiang District, Chengdu, Sichuan China; 4 West China School of Pharmacy, Sichuan University, Chengdu, Sichuan China; 5 Institute of Chinese Materia Medica, China Academy of Chinese Medical Sciences, Beijing, China

**Keywords:** Processing, Chinese herbal medicines, Decoction pieces, Standardization, Mechanism

## Abstract

Processing (*Paozhi*) represents a unique Chinese pharmaceutic technique to facilitate the use of Chinese herbal medicines (CHMs) for a specific clinical need in the guidance of Traditional Chinese Medicine (TCM) theory. Traditionally, most CHMs require a proper processing to meet the needs of specific clinical syndromes before being prescribed by TCM practitioners. During processing, significant changes in chemical profiles occur, which inevitably influence the associated pharmacological properties of a CHM. However, although processing is formed in a long-term practice, the underlying mechanisms remain unclear for most CHMs. The deepening understanding of the mechanism of processing would provide scientific basis for standardization of processing. This review introduced the role of processing in TCM and several typical methods of processing. We also summarized the up-to-date efforts on the mechanistic study of CHM processing. The processing mechanisms mainly include the following aspects: (i) directly reducing contents of toxic constituents; (ii) structural transformation of constituents; (iii) improving solubility of constituents; (iv) physically changing the existing form of constituents; (v) and influence by excipients. These progress may give new insights into future researches.

## Background

Processing, *Paozhi* in Chinese, is an ancient Chinese pharmaceutic technique to facilitate the use of Chinese herbal medicines (CHMs) for a specific clinical need in the guidance of Traditional Chinese Medicine (TCM) theory [1]. Processing of CHMs develops along with the history of TCM and promotes the formation of TCM theory in long-term practice, even wine serves as part of the ancient Chinese character ‘medicine’ for all its important role. Most CHMs need to be elaborately processed to become decoction pieces prior to their final consumption in the clinic or manufacture of proprietary drugs [2]. Processing represents a unique Chinese pharmaceutic approach that differentiates CHMs from other medicinal herbs in the world. In Chinese Pharmacopoeia (CP, 2015 edition), decoction piece(s) and related processing method(s) are clearly listed as a specific item of a CHM, and some decoction pieces like Astragali Radix Preparata Cum Melle are recorded as a separate CHM with independent quality control standards and indications [3]. In contrast, only few processed medicinal herbs and processing methods are recorded in the pharmacopoeias of other countries [4].

**Fig. 1:**
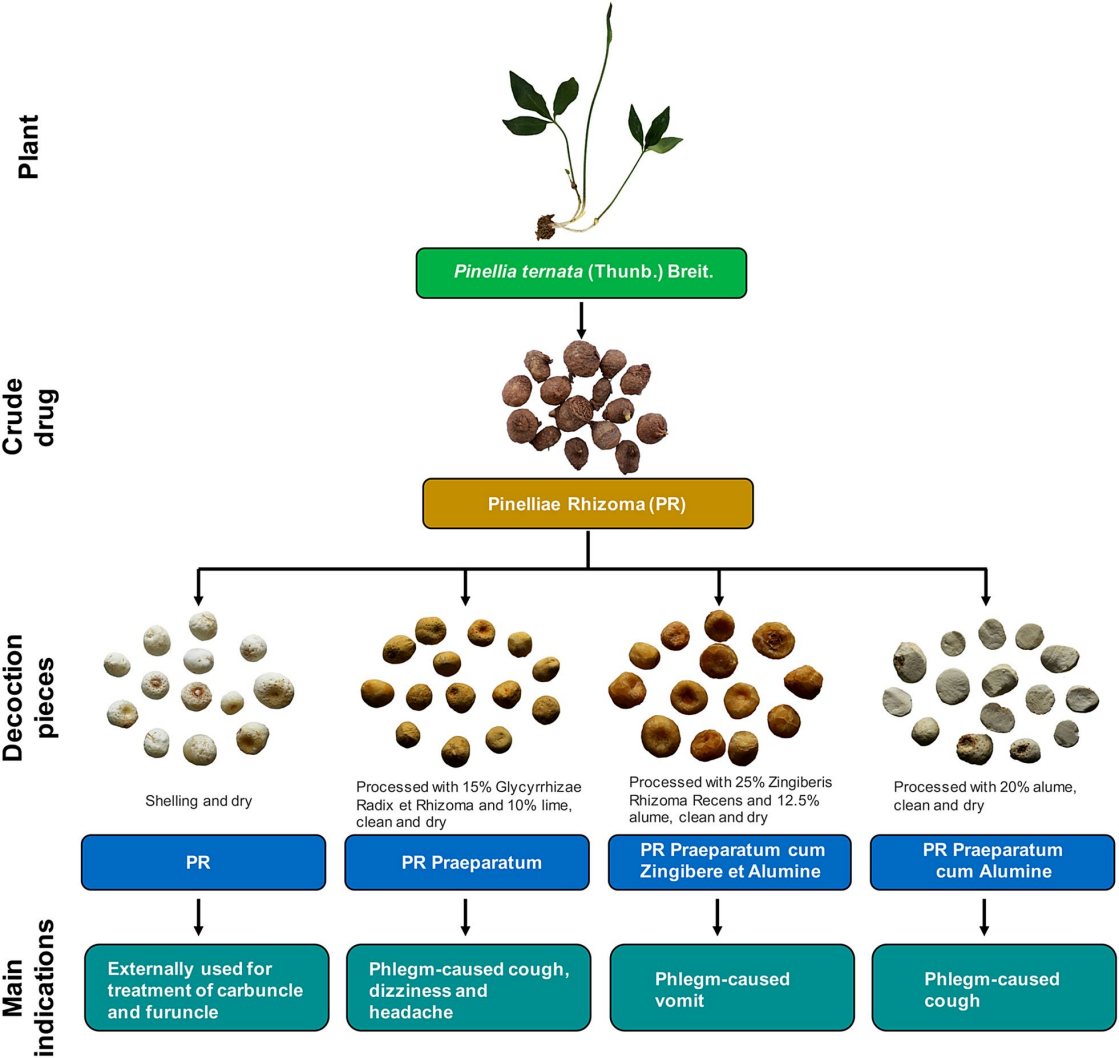
Four decoction pieces of PR recorded in the latest CP, as well as their respective processing methods and indications in clinic

Processing encompasses a series of techniques such as cutting, crushing, roasting, baking, and stir-frying with or without liquid/solid excipient, by which decoction pieces with different therapeutic potency can be derived from the same herb [1]. For instance, Pinelliae Rhizoma (PR) is a commonly used CHM for the treatment of phlegm-induced cough, vomit and headache [5]. Four processed PR are recorded in the latest CP, namely raw PR, PR Praeparatum (PRP, processed with 15% Glycyrrhizae Radix et Rhizoma and 10% lime), PR Praeparatum cum Zingibere et Alumine (PRZA, processed with 25% Zingiberis Rhizoma Recens and 12.5% alume) and PR Praeparatum cum Alumine (PRPA, processed with 20% alume) [3]. These decoction pieces produced by different processing methods are developed to reduce the toxicity of PR [6] and to guide and concentrate its therapeutic effects. Raw PR is often externally used for treatment of carbuncle and furuncle, PRP is inclined to relieve phlegm-caused cough, dizziness and headache, while PRZA and PRPA are respectively prescribed for phlegm-caused vomit and cough (Fig. 1).

Generally, processing can reduce toxicity, reinforce efficacy, alter energetic nature and therapeutic direction, as well as improve flavor of CHMs, thereby increase the therapeutic effectiveness and applicability of CHMs in individualized treatment. However, despite the extensive use of processed CHM, the underlying mechanisms of processing remain unclear for most CHMs to date. During the processing, particularly under heating and/or moist conditions, complicated changes in herbal components of CHMs may occur: the contents be increased or decreased; structures be changed; and/or novel compounds be formed. In many cases, the contents and structures of constituents may be altered simultaneously. Along with these changes mediated by processing, the pharmacological activity of a certain CHM may be changed accordingly. Therefore, investigation of the chemical and pharmacological changes of CHM before and after processing is key for understanding of underlying mechanisms. In the past few decades, emerging studies have been carried out to elucidate the mechanisms of processing. Herein, this review summarizes the up-to-date knowledge on these aspects, aiming to provide new insights to future researches.

## Methods of processing

The first recordation of processing can be dated back to 200 BC in Recipes for 52 Ailments (*Wushi’er Bingfang*), in which some classical methods like burning, calcining, stewing, and soaking were listed [7]. In the Northern and Southern dynasties, Master Lei’s discourse on processing (*Leigong Paozhi Lun*) appeared as the earliest book that systemically described the principles and methods of processing [8]. Afterwards, there are a series of monographs of processing that record and summarize the experiences of TCM practitioners. In broad terms, processing describes every procedure involved in preparing raw plants (or animal or mineral) into decoction pieces. In this review we mainly discuss these specific methods applied when the CHMs are cleaned, cut, and dried. Some commonly used processing methods are described below and listed in Table 1.

**Table 1:** Typical processing methods and representative processed CHMs listed in CP (2015 edition)

Processing method	Excipient	Representative processed CHM
Stir-frying (清炒)	–	Stir-fried Ziziphi Spinosae Semen (炒酸棗仁)
		Charred Crataegi Fructus (焦山楂)
		Carbonized Rhei Radix Et Rhizoma (大黃炭)
Stir-frying with liquid excipients (炙)	Yellow rice wine (酒)	Wine-fried Rhei Radix Et Rhizoma (酒大黃)
	Vinegar (醋)	Vinegar-fried Curcumae Rhizoma (醋莪術)
	Salt water (鹽)	Salt-fried Alpiniae Oxyphyllae Fructus (鹽益智仁)
	Fresh ginger juice (薑)	Ginger-fried Magnoliae Officinalis Cortex (薑厚樸)
	Refined honey (蜜)	Honey-fried Astragali Radix (炙黃芪)
Stir-frying with solid excipients (炒)	Wheat bran (麥麩)	Bran-fried Dioscoreae Rhizoma (麩炒山藥)
	Rice (米)	Rice-fried Mylabris (米斑蝥)
	River sand (砂)	Zingiberis Rhizoma Praeparatum (sand-fried Zingiberis Rhizoma 炮薑)
Steaming (蒸)	–	Ginseng Radix Et Rhizoma Rubra (Steamed Ginseng 紅參)
	Yellow rice wine (酒)	Wine-steamed Corni Fructus (酒萸肉)
	Vinegar (醋)	Vinegar-steamed Schisandrae Chinensis Fructus (醋五味子)
Boiling (煮)	Zingiberis Rhizoma Recens and alume	Arisaematis Rhizoma Preparatum (制天南星)
	Glycyrrhizae Radix et Rhizoma and lime	Pinelliae Rhizoma Praeparatum (法半夏)
Stewing (煨)	Straw paper (草紙)	Straw paper-stewed Aucklandiae Radix (煨木香)
	Wheat bran (麥麩)	Wheat bran-stewed Myristicae Semen (麩煨肉豆蔻)
Water trituration (水飛)	–	Water-triturated Cinnabaris (朱砂粉)
Calcining (煅)	–	Calcined Haematitum (煅赭石)

### Stir-frying

Cleaned and cut crude CHMs are fried in a pot, with or without the aid of excipients, while being constantly stirred until a certain degree of frying is obtained.

#### Stir-frying without excipients

Usually there are three degrees of stir-frying evaluated by the color in appearance and/or odor of a specific herb: stir-frying till yellow, till charred, and till carbonized (black outside and charred inside). Crataegi Fructus is a typical CHM that can be stir-fried until different degrees for distinct therapeutic purpose [9]. Un-processed Crataegi Fructus can promote digestion and invigorate blood circulation while stir-fried Crataegi Fructus is mainly used for indigestion. In contrast, charred Crataegi Fructus and carbonized Crataegi Fructus are used for the treatment of indigestion-caused diarrhea and gastrointestinal hemorrhage, respectively.

#### Stir-frying with liquid excipients

In order to reinforce and/or guide the efficiency of the herbs, many liquid excipients like yellow rice wine, vinegar and honey are often added to the crude herbs prior to stir-frying. For instance, processing with wine can enhance the effect of Angelicae Sinensis Radix in invigorating blood circulation [10], and wine-fried Angelicae Sinensis Radix is widely prescribed in many famous TCM formulae including Danggui Buxue decection, Siwu Decoction and Longdan Xiegan Pills.

#### Stir-frying with solid excipients

Similar to liquid excipient-assisted stir-frying, stir-frying with solid excipients also helps to extend the utility of CHMs. Stir-frying with rice represents an important approach of TCM practitioners to reduce the toxicity of some poisonous CHMs such as Mylabris [11] and reinforce the effect of many spleen-tonifying CHMs including Codonopsis Radix [12].

### Steaming

Steaming is a commonly used processing method to alter the properties of various CHMs by steaming the crude herbs with or without additional excipients. For example, steaming raw Polygoni Multiflori Radix with black bean juice can turn the anti-malarial and defecating effects to tonifying effects like liver and kidney replenishing, hair blackening, and bone strengthening [13, 14].

### Boiling

Boiling CHMs in water or in a herbal decoction can also (i) minimize the side effect of CHMs, such as Glycyrrhizae Radix decoction boiled Polygalae Radix to reduce the irritation on throat [15]; or (ii) enhance the therapeutic effect, such as vinegar boiled Curcumae Rhizoma to reinforce the effect in removing blood stasis.

### Stewing

Wrapping CHMs in moistened papers, bran or mud, and heating until the envelop becomes cracked or charred is another approach to reduce the undesired constituents and reinforce the astringent effect of CHMs. Wheat bran-stewed Myristicae Semen is the major form of Myristicae Semen in clinical application due to reduced irritant oils [16]. Stewing using moistened straw paper endows Aucklandiae Radix with stronger astringent property and enhance the anti-diarrhea effect [17].

### Other processing methods

Many other methods are widely applied to guarantee the safety and effectiveness of CHMs. For instance, water trituration is a repetitious and complicated process by grounding mineral CHMs with water to obtain extremely fine powder. Many mineral and crustaceous CHMs can be calcined directly or indirectly in the flames to render these hard CHMs crisp and thus easy to crush.

## Advances in understanding the mechanism of processing

**Fig. 2:**
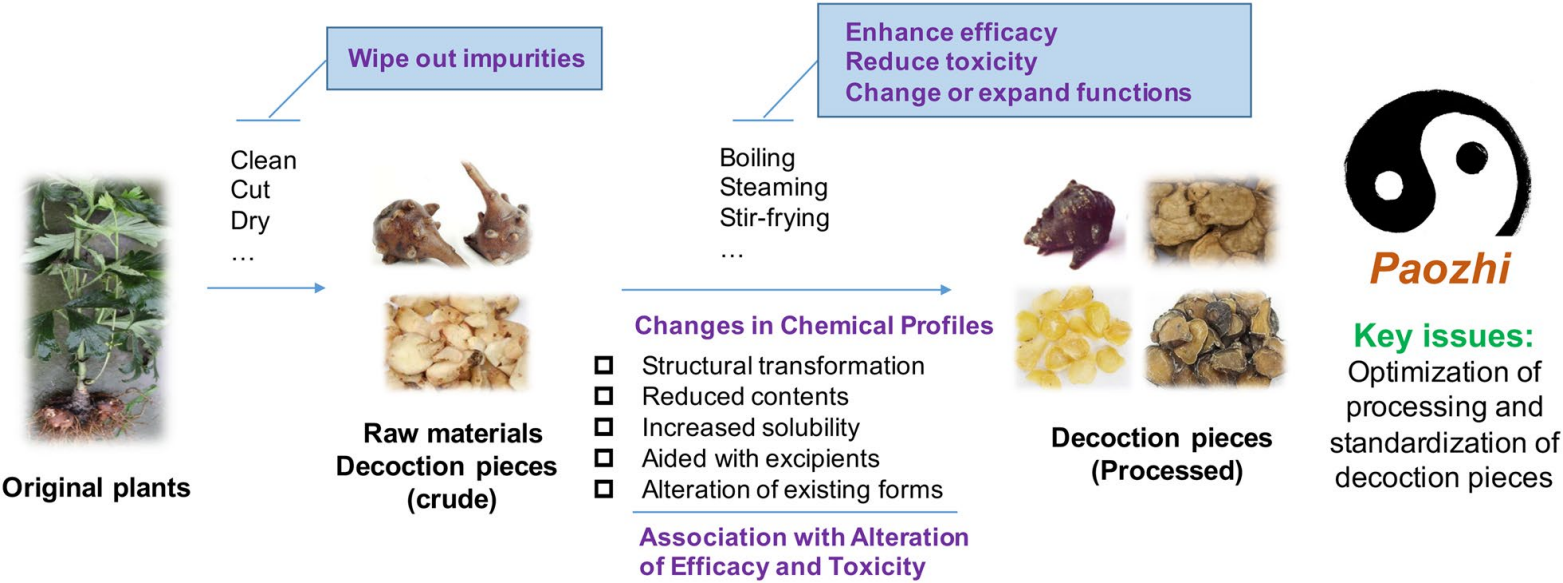
Understanding of traditional aspects of CHM processing (*Paozhi*) via advanced chemical and pharmacological evaluations. *Paozi* results in complex changes in chemical profiles of CHMs via structural transformation, reduced contents, increased solubility, alteration of existing form of constituents and influence by excipients. Inevitably, these chemical changes lead to alteration of efficacy and/or toxicity of CHMs. *Paozi* can adjust the nature (heat, warm, cold and cool) of a certain CHM to facilitate the symptomatic and accurate prescription by TCM practitioners and help equilibrate the balance between Yin and Yang in human body. As a traditional technique, the key issues in modernization of *Paozi* are the optimization of processing method and the standardization of decoction pieces. The processing of Aconitum root is illustrated as a representative

Processing is an important feature of CHM, which is formed early in the history of TCM and has developed along with its clinical practice. The methods and purposes of processing are usually different for different herbs, while processing might have multiple influences on a certain herb. In TCM theory, disease is often a result of imbalance between Yin and Yang in human body. It is believed that processing can adjust the nature (heat, warm, cold and cool) of a certain CHM to facilitate the symptomatic and accurate prescription by TCM practitioners and help equilibrate the balance between Yin and Yang in human body. In this regard, traditionally, most CHMs require proper processing before being prescribed. Processing may directly reduce the contents of toxic constituents, transform the structure of constituents, or increase the solubility of active constituents (Fig. 2). Efforts have been made in recent years to understand the traditional aspect of processing. Some representative evidences in elucidating the mechanisms of CHM processing are displayed in Table 2.

**Table 2:** Mechanisms of processing of representative CHMs

Decoction pieces		Purpose and major mechanisms of processing	References
Crude CHM	Processed CHM (processing method)		
Aconiti Radix, Chuanwu 川烏	Aconiti Radix Cocta, Zhichuanwu 制川烏 (soaking, boiling or steaming)	*Purpose* Reducing toxicity *Mechanisms* Structural transformation of toxic constituents: (1) highly toxic diester diterpene alkaloids hydrolyze or decompose into monoester diterpene alkaloids of low toxicity or non-toxic non-esterified diterpene alkaloids. (2) Diester diterpene alkaloids react with components in Glycyrrhizae Radix to generate lipo-alkaloids of low-toxicity. On the other hand, the resultant alkaloids have considerable anti-inflammatory and analgesic effects	[18–20]
Aconiti Lateralis Radix, Nifuzi 泥附子	Aconiti Lateralis Radix Praeparata, Yanfuzi 鹽附子 (soaking)		
	Aconiti Lateralis Radix Praeparata, Danfupian 淡附片 (soaking in salt water, boiling with Glycyrrhizae Radix and black bean)		
	Aconiti Lateralis Radix Praeparata, Heishunpian 黑順片 (soaking in salt water, staining and steaming)		
	Aconiti Lateralis Radix Praeparata, Baifupian 白附片 (soaking in salt water, peeling and steaming)		
	Paofupian炮附片 (sand-scorch of Heishunpian or Baifupian)		
Aconiti Kusnezoffii Radix, Caowu 草烏	Aconiti Kusnezoffii Radix Cocta, Zhicaowu 制草烏 (soaking in water and boiling)		
Pinelliae Rhizoma, Banxia 半夏	Pinelliae Rhizoma Praeparatum, Fabanxia 法半夏 (soaking with water and then with Glycyrrhizae Radix juice)	*Purpose* Reducing toxicity *Mechanisms* (1) Physically changed crystal structure: alum solution changes the structure of needle-like calcium oxalate crystals and dissolves the lectin in the crystals, which decreases the side effect. (2) Detoxifying components from excipients: a compound gingerol from ginger juice can effectively inhibit Banxia-induced inflammation	[21–25]
	Pinelliae Rhizoma Praeparatum Cum Zingibere et Alumine, Jiangbanxia 姜半夏 (soaking with water, boiling with ginger and alum)		
	Pinelliae Rhizoma Praeparatum Cum Alumine, Qingbanxia 清半夏 (soaking with alum solution)		
Typhonii Rhizoma, Baifuzi 白附子	Zhibaifuzi 制白附子 (soaking with alum solution)		
Rhei Radix et Rhizoma, Dahuang 大黃	Jiudahuang 酒大黃 (stir-frying with alcohol)	*Purpose* Changing functions and reducing toxicity *Mechanisms* (1) Decomposing of conjugated anthraquinones into the corresponding free anthraquinones; (2) reduced contents of tannins; (3) after processing, Dahuangtan has no effect on blood circulation	[26–28]
	Shudahuang 熟大黃 (steaming or steaming with alcohol)		
	Dahuangtan 大黃炭 (charring)		
Angelicae Sinensis Radix, Danggui 當歸	Jiudanggui 酒當歸 (stir-frying with alcohol)	*Purpose* Enhancing efficacy *Mechanisms* (1) Increasing the solubility of ferulic acid; (2) decreasing the content of Z-ligustilide. Both ferulic acid and Z-ligustilide are biological constituents, but high concentration of Z-ligustilide is irritant	[10, 29–31]
Ginseng Radix et Rhizoma, Renshen 人參	Ginseng Radix et Rhizoma Rubra, Hongshen 紅參 (steaming)	*Purpose* Enhancing efficacy and reduced side effect *Mechanisms* (1) Structural transformation of ginsenosides via hydrolysis of sugar moieties and/or epimerization of 20(S)-type into 20(R)-type; (2) Maillard reaction on reducing sugars and amino acids to form phenol compounds; (3) degradation of dencichine which has neurotoxicity. These changes contribute to enhanced anti-oxidant, anti-cancer and immue-modulating effects, and reduced side effect	[32–37]
Strychni Semen, Maqianzi 馬錢子	Zhimaqianzi 制馬錢子 (stir-frying with sand)	*Purpose* Reducing toxicity *Mechanisms* Decomposition and oxidation of highly-toxic strychnine and brucine to generate isostrychnine, isobrucine, brucine N-oxide and strychnine N-oxide	[38–41]
Mylabris, Banmao 斑蝥	Mibanmao 米斑蝥 (stir-frying with rice)	*Purpose* Reducing toxicity *Mechanisms* Reducing contents of toxic constituents: stir-frying of Banmao facilitates sublimation of cantharidin when the processing temperature reaches 120 °C, and the content of cantharidin is significantly reduced	[42]
Crotonis Fructus, Badou 巴豆	Crotonis Semen Pulveratum, Badoushuang 巴豆霜 (partially removal of croton oil)	*Purpose* Reducing toxicity *Mechanisms* Reduced contents of toxic constituents: processing via removal of Crotonis oil which contains toxic constituents reduces toxicity of Badou	[43]
Atractylodis Macrocephalae Rhizoma, Baizhu 白術	Fuchaobaizhu 麩炒白術 (stir-frying with bran)	*Purpose* Enhancing efficacy *Mechanisms* Structural transformation via decomposing atractylone into Atractylenolide I and II during processing	[44, 45]
Genkwa Flos, Yuanhua 芫花	Cuyuanhua 醋芫花 (stir-frying with vinegar)	*Purpose* Reducing toxicity and enhancing efficacy *Mechanisms* (1) The contents of Yuanhuacine and genkwadaphnin which are highly toxic are decreased; (2) the contents of bioactive flavonoids, including genkwanin, 3′-hydroxy-genkwanin and apigenin, are increased, likely due to the transformation of flavonoid glycosides into the respective glycones	[46]
Glycyrrhizae Radix et Rhizoma, Gancao 甘草	Glycyrrhizae Radix et Rhizoma Praeparata Cum Melle, Zhigancao 炙甘草 (stir-frying with honey)	*Purpose* Enhancing efficacy *Mechanisms* Hydrolysis of glycosides such as glycyrrhizin, liquiritin apioside and isoliquiritin apioside into glycyrrhetinic acid, liquiritigenin and isoliquiritigenin, respectively, with enhanced anti-inflammatory effect	[47]
Calamina, Luganshi 爐甘石	Duanluganshi 煆爐甘石 (calcining)	*Purpose* Enhancing efficacy *Mechanisms* Decomposing ZnCO3 into ZnO which has better antimicrobial activity	[48, 49]
Leaves of *Baphicacanthus cusia* (Nees) Bremek., *Polygonum tinctorium* Ait. or *Isatis indigotica* Fort.	Indigo Naturalis, Qingdai 青黛	*Purpose* Enhancing efficacy *Mechanisms* Decomposing isatan B or indole glycoside and further condensed to form indigos and indirubin, the active constituents	[50]
Kansui Radix, Gansui 甘遂	Cugansui 醋甘遂 (stir-frying with vinegar)	*Purpose* Reducing toxicity *Mechanisms* (1) Conversion of the highy-toxic 3-Acyl ester components into the non-toxic 20-acyl ester components; (2) reaction of diterpenes with acetic acid to form acetylated diterpenes with poor solubility which decreases toxicity	[51, 52]
Sinapis Semen, Jiezi 芥子	Chaojiezi 炒芥子 (Stir-frying)	*Purpose* Reducing side effect *Mechanisms* Inactivation of myrosase via heating to retain the glucosinolates, including sinalbrin	[53]
Xanthii Fructus, Cang’erzi 蒼耳子	Chaocang’erzi 炒蒼耳子 (stir-frying)	*Purpose* Reducing toxicity *Mechanisms* Decomposing β-d-Fructofuranosyl-α-d-glucopyranoside and other glycosides	[54]
Epimedii Folium, Yinyanghuo 淫羊藿	Zhiyinyanghuo 炙淫羊藿 (stir-frying with mutton fat)	*Purpose* Enhancing efficacy *Mechanisms* Decomposing flavonoid glycosides to form secondary glycosides or aglycones, which results in enhanced gonadal function	[55, 56]
Coptidis Rhizoma, Huanglian 黃連	Jiuhuanglian 酒黃連 (stir-frying with alcohol)	*Purpose* Enhancing efficacy *Mechanisms* (1) Increased solubility of the contents of berberine, palmatine, coptisine and jatrorrhizine; (2) decomposing of berberine to form a novel compound berberubine which has anticancer activity	[57, 58]

### Directly reducing contents of toxic constituents

The primary concept of detoxification is to reduce the contents of toxic constituents in CHM. Processing has been proved as a useful means to reduce the toxicity of certain CHMs. Toxic compounds usually possess unique physical characteristics. Based on this, specific processing methods may efficiently reduce their contents in the corresponding CHMs.

Mylabris (Banmao), is derived from the blister beetles *Mylabris phalerata* Pallas or *M. cichorii* Linnaeus, and is a famous poisonous CHM using for treating cancers [59, 60]. The internal use of Banmao often leads to serious nephrotoxicity which is lethal [61]. Traditionally, Mylabris is stir-frying processed with or without the presence of rice. In recent years, Mylabris is also processed with sodium hydroxide solutions. Both methods have been proved to reduce its toxicity [62]. It has been demonstrated that cantharidin, a terpenoid defensive toxin, is responsible for the therapeutic action as well as toxicity of Mylabris [63–65]. Therefore, control of the contents of cantharidin is key for safe and effective use of Mylabris. A number of studies show that cantharidin can be readily sublimated when the processing temperature reaches 120 °C, and thus its contents in raw materials are significantly reduced [66]. Furthermore, in alkaline condition of sodium hydroxide solution, cantharidin becomes the form of cantharidinate sodium, which is less nephrotoxic than the original form [67, 68]. Based on these findings, different processing methods result in the decreased contents of highly-toxic cantharidin and thus reduce the toxicity of Mylabris.

Crotonis Semen (Badou, in Chinese) is the dried fruit of *Croton tiglium* L., and is used in TCM for treatment of ascites, constipation, diphtheritis, acute laryngitis and laryngeal obstruction [69]. Raw Crotonis Semen is highly toxic and can cause hemolysis and severe diarrhea. It is demonstrated that the toxic components mainly exist in the Croton oil [70, 71]. Traditional processing method to remove oil from Crotonis Semen can remarkably reduce the contents of toxic constituents, resulting in reduced toxicity.

### Structural transformation of constituents

Many methods of processing, such as stir-frying, steaming and boiling, necessitate the heating and/or moist conditions, which inevitably leads to complex chemical changes in processed CHMs. Structural transformation of herbal components is one of the most common consequences due to processing. Herbal components may undergo oxidation, decomposition, isomerization, hydrolysis and/or reaction with other constituents, eventually, to form novel compounds [72]. This often results in alteration of pharmacological or toxicological properties of processed CHMs compared to the raw ones. Some of CHMs, including the Aconitum root, Ginseng Radix et Rhizome and Rhei Radix et Rhizoma, have been demonstrated to possess distinct chemical profiles after processing and show reduced toxicity or altered therapeutic activities.

#### Aconitum root: decomposing of highly toxic components during processing leads to detoxification

Chuanwu (*Aconiti* Radix, the mother root of *A. carmichaeli*), Fuzi (*A. Lateralis* Radix, the daughter root of *A. carmichaeli*) and Caowu (*A. kusnezoffii* Radix, the root of *A. kusnezoffii*) are three most popular Aconitum herbs used in TCM and are documented in the latest CP [73, 74]. Raw Aconitum plants are extremely dangerous, and can only applied in external use. They are used in decoction, proprietary medicines and other formulations only after being properly processed (repeated boiling or steaming). Aconitum root induces remarkable cardiotoxicity and neurotoxicity. The toxidrome of acute aconite poisoning is a combination of cardiovascular, neurological, gastrointestinal and other symptoms [75]. Despite their toxicity and narrow therapeutic window, Aconitum root has been widely used in TCM due to their anti-inflammatory, analgesic and cardiotonic properties [76]. Till now, there are six different types of processed Aconitum medicinals, including Zhichuanwu, Yanfuzi, Danfupian, Heishunpian, Baifupian and Zhicaowu, which are documented in the latest CP. Regardless of the distinct processing methods, many researches have demonstrated that properly processed Aconitum root showed reduced toxicity [77, 78].

The toxicity of Aconitum herbs is mainly due to the presence of Aconitum alkaloids at high concentrations [79, 80]. These alkaloids have been found to target voltage-sensitive sodium channels in myocardium, nerves and muscles, and cause cardiotoxicity and neurotoxicity [81, 82]. C_19_-diterpenoid-type alkaloids are found to be the main constituents of aconitum [73]. These alkaloids are further classified into four types: diester diterpenoid alkaloids (DDA), such as aconitine, mesaconitine, and hypaconitine; monoester diterpenoid alkaloids (MDA), such as benzoylaconine, benzoylhypaconine, and benzoylmesaconine; non-ester diterpenoid alkaloids (NDA), such as aconine, mesaconine, and hypaconine; and lipoalkaloids. A series of studies have demonstrated that the DDA can be decomposed into MDA by losing an acetic acid at C-8 position during processing, which further undergo elimination of a benzoylic acid at C-14 position to generate NDA, or substitution with a fatty acid acyl group at C-8 position to form lipoalkaloids [18–20]. For instance, at the heating and moist condition (boiling or steaming), aconitine, mesaconitine and hypaconitine could be firstly converted into benzoylaconine, benzoylmesaconine and benzoylhypaconine, respectively, and further transformed into aconine, mesaconine, and hypaconine, respectively [83, 84]. After processing, the contents of the DDA (aconitine, mesaconitine and hypaconitine) were significantly reduced in Fuzi [84]. Since DDA are much toxic (100- to 400-fold) than MDA and lipoalkaloids, decomposing of DDA has been identified as the main mechanism for detoxification of aconitum processing [73]. Notably, MDA and lipoalkaloids also display remarkable anti-inflammatory and analgesic effects.

Traditionally, the processing of Aconitum root is monitored by tasting the spicy flavor which should gradually fade to certain extent. With the understanding of the underlying mechanisms, processing of aconitum is now controlled by determination of the marker alkaloids. For instance, as recorded in the latest CP, the total contents of DDA-type constituents should not be higher than 0.02% (g/g), while the contents of NDA-type constituents should be no less than 0.01% (g/g).

#### Ginseng: structural transformation of ginsenosides during processing results in enhanced efficacy

Ginseng Radix et Rhizome (Renshen, in Chinese) has been traditionally used in TCM for thousands of years, and is also one of the most popular functional food in Asian countries [85, 86]. Ginsenosides, the triterpene saponins, have been found to be the main bioactive constituents in ginseng, which are responsible for anti-oxidant, antidiabetic, immune modulatory, anti-inflammatory and anti-cancer properties [87–89]. Their structures are mainly grouped into dammarane type with 20(S)-protopanaxadiol and 20(S)-protopanaxatriol as the aglycone and oleanane type [90].

White ginseng (the fresh ginseng air-dried) and the processed one, Hongshen (the fresh ginseng steamed for 2–3 h and dried), are two types of ginseng products available in the market. Traditionally, Hongshen is considered to be more powerful in “boosting yang” than the White ginseng [91, 92]. Several reports have suggested that certain activities of Hongshen are better than the White ginseng [93]. During processing (steaming), complex chemical changes occur in terms of ginsenosides. The malonyl-ginsenosides, which are only found in the white ginseng, are de-malonylated and converted into the corresponding ginsenosides [94, 95]. The sugar chains at C-20 and/or C-3 are further hydrolyzed [95]. Furthermore, the 20(S)-type ginsenosides can be transformed into 20(R)-type [90, 94, 95]. As a result, the chemical profile of White ginseng and Hongshen are considerably different. The polar ginsenosides in White ginseng becomes the less polar ones. The characteristic ginsenosides in Hongshen include 20(S)-, 20(R)-Rg_3_, Rk_3_, Rh_4_, Rk_1_, Rg_5_, etc., which have been demonstrated to exhibit more potent anti-cancer, anti-diabetic, and anti-inflammatory effects [96, 97]. Therefore, structural transformation of ginsenosides during processing results in enhanced efficacy of the steamed ginseng.

### Improved solubility of active constituents

Emerging evidences indicate that processing improves the solubility of herbal constituents in certain CHMs. Under heating condition, excipients used in processing such as wine and vinegar often help active constituents more easily to dissolve from a complex texture. Eventually, the processed CHMs show enhanced efficacy.

Coptidis Rhizoma (Huanglian, in Chinese) is derived from the dried rhizome of *Coptis chinensis* Franch., *C. deltoidea* C. Y. Cheng et Hsiao or *C. teeta* Wall, and is traditionally used for toothache, dysentery, hypertension, inflammation and liver diseases [98, 99]. Alkaloids, such as berberine, palmatine, epiberberine and coptisine, are found to be one of the main types of active constituents [100]. It is reported that the dissolution rate of total alkaloids in wine-processed Coptidis Rhizoma reaches 90%, while that in raw medicinals is only 58%. After processing, the contents of berberine, palmatine, coptisine and jatrorrhizine that were detected in the processed Coptidis Rhizoma were significantly increased [57]. This observation is also seen on Angelicae Sinensis Radix (Danggui, in Chinese). Danggui, the dried root of *Angelica sinensis* (Oliv.) Diels., is a famous CHM and has been used for more than 2000 years in China as a dietary supplement for women’s health [10]. A recent study showed that yellow wine-processed Danggui displays a significant increase in solubility of ferulic acid, one of the major biological components [10].

### Physically changing the existing form of constituents

Processing can also change the existing form of constituents in CHMs, which may influence their actions. One example is the PR, the dried tuber of *P. ternata* (unb.) Breit. It is first recorded in Shen-Nong-Ben-Cao-Jing (Shen Nong’s Herbal Classic, B.C. 100–200), and is widely used in TCM to treat cough, phlegm, vomiting and cancer [25, 101]. Similar to the Aconitum, raw PR is very toxic and can be only applied for external use. In order to reduce its toxicity, alum solution is always used in the processing of PR. Recent studies showed that aluminium ions in the alum solution were capable of complexing with oxalic acid in calcium oxalate of raphides, which helped to dissolve calcium oxalate and thus altered the unique rigid crystal structure [24]. This further led to the dissolve and degradation of the lectin inside the raphides [24]. As a result, the pro-inflammatory effect of raphides was significantly decreased. Therefore, physically structural alteration of needle-like calcium oxalate crystals contributes to the reduction of toxicity of PR during processing.

### Influences of excipients

Excipients, including wine, vinegar, ginger juice, honey, rice, Glycyrrhizae Radix et Rhizoma, Euodiae Fructus and mutton fat, are frequently used in processing of CHMs to meet different purposes, and sometimes play an important role. Wine, vinegar and honey are commonly used as solvents to promote the solubility of several types of naturally-occurring compounds. As discussed above, wine can help the dissolve of active constituents of Danggui and Huanglian [10, 57]. Meanwhile, some excipients can react with the constituents in specific CHMs. For instance, during vinegar-assisted processing the toxic diterpenes in Kansui Radix (Gansui) can react with acetic acid to form acetylated diterpenes with poor solubility, which results in reduced toxicity [51, 52].

Notably, some excipients themselves, such as Glycyrrhizae Radix et Rhizoma, Euodiae Fructus and honey, are derived from CHMs and have their own therapeutic effects. Several studies show that constituents from these excipients are important for reducing toxicity and/or enhancing efficacy. As above described, 25% juice of Zingiberis Rhizoma Recens is used in the processing of PR Praeparatum cum Zingibere et Alumine (Jiangbanxia). It is demonstrated that gingerol derived from the ginger juice can remarkably inhibit Banxia-induced inflammation, which contributes to the detoxification effect [102]. Euodiae Fructus (Wuzhuyu) is the dried fruit of *E. rutaecarpa* (Juss.) Benth., *E. rutaecarpa* (Juss.) Benth. *var. officinalis* (Dode) Huang, or *E. rutaecarpa* (Juss.) Benth. *var. bodinieri* (Dode) Huang, and its processed products are produced by boiling raw materials with Glycyrrhizae Radix [103, 104]. Studies have shown that Glycyrrhizae Radix can enhance the analgesic effects of Wuzhuyu. After processing, the content of hydroxyevodiamine is reduced significantly, while that of evocarpine is increased [105].

## Conclusion and future perspectives

Processing is formed in long-term practice with a systematic theory, and represents one of the therapeutic wisdoms of TCM. Since most crude materials of CHMs require proper processing before being used, standardization of processing is a prerequisite for standardization of CHM. However, it is of much difficulty in terms of this aspect. Firstly, the methods of processing vary significantly in different regions of China [7]. For certain CHMs, there is no unified processing practice for all areas of China. Although there are a total of 618 decoction pieces that have been adopted in the latest CP, a large number of processed CHMs are not covered. Most CHMs recorded in the local standards of different provinces have used different methods [106]. The use of excipients also sometimes varies [106]. Secondly, even in the latest CP, the processing practice is not accurately described. It is reported that the bioactive or toxic constituents can be changed over time and processing temperature [107–109]. The use of excipients is also important. For instance, different types and concentration of wine have distinct impact on the main compositions and contents of the alkaloids of *Coptis chinensis* [110]. Notably, there is no standards for most excipients used. Based on these facts, it is difficult to control the procedure of processing in practice. Traditionally, pharmaceutical workers process CHMs mainly according to their experiences to judge the color, flavor or appearance of CHMs. In a recent study, Fei et al. analyzed the color values of the peel and flesh of Crataegi Fructus and constructed related mathematical functions to effectively evaluate the processing degree of Crataegi Fructus [9]. Some researchers have also suggested to use novel techniques such as microwaves, which can be easily controlled [111, 112]. However, whether these new evaluation systems or techniques are able to produce qualified products still needs more assessment before applying to industry. Till now, the efforts for optimization and standardization of processing are still largely needed.

Another challenge is the standardization of decoction pieces, especially the processed CHMs. At current stage, there are no quality control standards for most processed CHMs. As described in this review, there are complex chemical changes in processing which are usually associated with alterations in pharmacological effects. Therefore, the deepening understanding of the underlying mechanisms of processing is of great significance for the standardization of CHMs including the selection of markers.

Investigation of the mechanisms of processing has been ongoing for several decades. With the development of novel concepts, techniques and models, great advances have been achieved, although most parts of processing remain unclear. In this review, we have summarized current progress with regards of processing mechanisms into the following aspects: (i) directly reducing contents of toxic constituents; (ii) structural transformation of constituents; (iii) improving solubility of constituents; (iv) physically changing the existing form of constituents; (v) influence by excipients. Most studies have focused on changes in chemical profiles of processed CHMs. The application of new technologies such as NMR, GC–MS and LC–MS has greatly facilitated the qualitative and quantitative analysis of herbal constituents, even at trace concentrations [41, 113–115]. Due to the changed chemical profiles, the finding of chemical markers that are pharmacologically relevant is essential for evaluating the processing practice. Several studies have demonstrated that “omics” studies are efficient and may at least partially represent holistic perspectives [116–119]. In a recent report, targeted glycomics and untargeted metabolomics were used to investigate the overall chemical characterization of Rehmanniae Radix [116]. The obtained data were further processed by multivariate statistical analysis. Finally, the processing-induced chemical transformation was summarized to evoke the mechanism behind processing. In another study, metabolomics study revealed seven chemical markers of raw and processed Atractylodis Macrocephalae Rhizoma [118]. However, despite these advances, most studies do not investigate the association of chemical and pharmacological changes. It is always valuable to assess the contribution of alteration of chemical compositions and formation of novel compounds to changed bioactivities of a CHM.

As mentioned above, decoction pieces are the only form directly applied in clinical practices. However, many studies have used the raw herb, instead of the decoction pieces, for chemical and pharmacological evaluations, which do not take into consideration of the chemical changes during processing of CHMs. This would possibly or sometimes inevitably lead to bias in understanding the traditional use of CHMs. Therefore, it is essential to use decoction pieces, especially the processed ones, for modern CHM researches.

Taken together, standardization of processing methods of CHM is a prerequisite to maintain the quality and guarantee the safety of CHM. To set up unified and scientific processing practices of CHM, further efforts should be paid to elucidate the mechanism of processing using advanced and comprehensive technologies.

## Abbreviations

CHM: Chinese herbal medicine
CP: Chinese Pharmacopoeia
PR: Pinelliae Rhizoma
PRP: PR Praeparatum
PRZA: PR Praeparatum cum Zingibere et Alumine
PRPA: PR Praeparatum cum Alumine
TCM: Traditional Chinese Medicine
